# Removing Noises Induced by Gamma Radiation in Cerenkov Luminescence Imaging Using a Temporal Median Filter

**DOI:** 10.1155/2016/7948432

**Published:** 2016-08-25

**Authors:** Xu Cao, Yang Li, Yonghua Zhan, Xueli Chen, Fei Kang, Jing Wang, Jimin Liang

**Affiliations:** ^1^Engineering Research Center of Molecular and Neuro Imaging, Ministry of Education and School of Life Science and Technology, Xidian University, Xi'an, Shaanxi 710071, China; ^2^Department of Nuclear Medicine, Xijing Hospital, Fourth Military Medical University, Xi'an, Shaanxi 710032, China

## Abstract

Cerenkov luminescence imaging (CLI) can provide information of medical radionuclides used in nuclear imaging based on Cerenkov radiation, which makes it possible for optical means to image clinical radionuclide labeled probes. However, the exceptionally weak Cerenkov luminescence (CL) from Cerenkov radiation is susceptible to lots of impulse noises introduced by high energy gamma rays generating from the decays of radionuclides. In this work, a temporal median filter is proposed to remove this kind of impulse noises. Unlike traditional CLI collecting a single CL image with long exposure time and smoothing it using median filter, the proposed method captures a temporal sequence of CL images with shorter exposure time and employs a temporal median filter to smooth a temporal sequence of pixels. Results of in vivo experiments demonstrated that the proposed temporal median method can effectively remove random pulse noises induced by gamma radiation and achieve a robust CLI image.

## 1. Introduction

Cerenkov luminescence imaging (CLI) has the ability to optically visualize radioactive decay signals from medical isotopes using optical imaging instruments and attracts more and more attention. CLI utilizes a type of electromagnetic radiation called Cerenkov radiation produced when a charged particle travels faster than the speed of light through an insulating medium [1, 2]. CLI collects molecular information by detecting Cerenkov luminescence (CL) in a continuous spectrum from the ultraviolet through the visible spectrum emitted during Cerenkov radiation. CLI can offer Cerenkov radiations of many clinically available radionuclides for positron emission tomography (PET) and single photon emission computed tomography (SPECT) with relatively lower cost optical devices. Compared to PET and SPECT, CLI has the potential for bridging the information acquired from nuclear and optical imaging.

Robertson et al. first performed CLI on mice using positron-emitting radiotracers [3]. Furthermore, several research groups independently showed the feasibility of CLI for disease targeting and drug tracking in small animals [4–9]. Using an optical tomographic reconstruction method, researchers have obtained tomographic images using CL termed as Cerenkov luminescence tomography (CLT) [10–15]. Intraoperative or endoscopic imaging based on CL is also reported [16–19]. It is very encouraging that CLI has been used for clinical imaging of human body [20, 21].

However, the very weak CL from Cerenkov radiation of radionuclides is susceptible to high level of impulse noises introduced by high energy gamma rays. To obtain strong enough CL signals for imaging, traditional CLI collects one image of CL with long exposure time about several minutes. At the same time, a great number of gamma rays may reach the CCD sensor and produce heavy random impulse noises. So, using shorter exposure time is beneficial to reduce the random impulse noises induced by gamma radiation on the premise of obtaining observable CL signals.

In the current study, single CL image acquisition with minute-long exposure time is substituted by a temporal sequence of CL images with second-long exposure time to suppress these random impulse noises. Accordingly, a temporal median (TM) filter is proposed to remove the high level impulse noises induced by gamma radiation in CLI. Unlike traditional median filter smoothing pixels within spatial domain, TM filter smooths pixels within time domain, which is more effective to remove this kind of additive random noises.

## 2. Material and Methods

### 2.1. Temporal Median Filter

**Figure 1:**
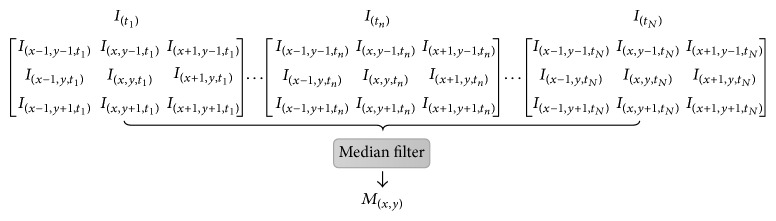
Schematic diagram of temporal median filter.

Figure 1 shows the scheme of TM filter. *N* CLI images are consecutively collected, and *I*_(*x*, *y*, *t*_*n*_)_ is the gray value at pixel location (*x*, *y*) for CLI image *I*_(*t*_*n*_)_ collected at *t*_*n*_ time point.

We select 9 pixels containing eight neighbor pixels of *I*_(*x*, *y*, *t*_*∗*_)_ and *I*_(*x*, *y*, *t*_*∗*_)_ itself for each CLI image collected at *t*_*∗*_ time point, and then 9*N* pixels for *N* CLI images are used to construct a 1-D vector:

(1) $$\begin{array}{c} U_{\left(x,y\right)}=\left[\;I_{\left(x-1,y-1,t_{1}\right)},\ldots ,I_{\left(x,y,t_{n}\right)},\ldots ,I_{\left(x+1,y+1,t_{N}\right)}\;\right]. \end{array}$$

To get the median of *U*_(*x*, *y*)_, we sort *U*_(*x*, *y*)_ in ascending order and obtain a new 1-D vector:

(2) $$\begin{array}{c} R_{\left(x,y\right)}=\left[\;r_{\left(x,y,1\right)},\ldots ,r_{\left(x,y,n\right)},\ldots ,r_{\left(x,y,9N\right)}\;\right], \end{array}$$

where *R*_(*x*, *y*)_ (*r*_(*x*, *y*, 1)_ ≤ ⋯≤*r*_(*x*, *y*, *n*)_ ≤ ⋯≤*r*_(*x*, *y*, 9*N*)_) are the elements of *U*_(*x*, *y*)_. The final filtered pixel value at location (*x*, *y*)  is the value at the middle position in *R*_(*x*, *y*)_:

(3) $$\begin{array}{c} M_{\left(x,y\right)}=r_{\left(x,y,\left\lceil 9N/2\right\rceil \right)}, \end{array}$$

where ⌈9*N*/2⌉ is the rounded up nearest integer of 9*N*/2. *N* is the number of CLI images for a temporal sequence, which also means the sequence length in time domain.

### 2.2. Numerical Simulation

**Figure 2:**
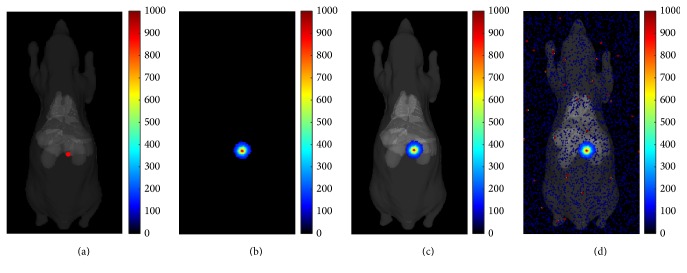
Numerical simulation of CL image. (a) The digital mouse and CL source location. (b) The simulated CLI image. (c) The fusion image of simulated CL and CT data. (d) The fusion image of simulated CL with impulse noises and CT data.

A Monte Carlo simulation using MOSE software based on digital mouse atlas is conducted to get the CL image. A point light source mimicking CL source generated from medical isotopes is subcutaneously embedded on back of the digital mouse with the depth about 5 mm as shown in Figure 2(a). The optical parameters *μa* and *μs* of each organ under the wavelength of 620 nm are adopted from [22].  Figure 2(b) is the simulated CL image, and Figure 2(c) is the fused image with mouse atlas. Two levels of random noises were added into the CL image to simulate dark noises of CCD with low values and impulse noises induced by gamma radiation with high values (Figure 2(d)).

### 2.3. Materials

^18^F-FDG was provided from the Department of Nuclear Medicine, Xijing Hospital, Fourth Military Medical University. Animal was cared for in accordance with a protocol approved by the Xidian University Animal Care and Use Committee. A Kunming mouse with abdomen unhairing using depilatory cream was used as the imaging object. A pseudotumor was provided using 100 uCi of ^18^F-FDG with the volume 20 uL mixed with 20 uL matrigel (BD Biosciences, Sparks, MD) injected to abdomen of the mouse.

### 2.4. Optical Imaging

All CLI images were acquired using a home-made in vivo animal optical imaging system, which includes an Andor Ixon Ultra 897 EMCCD with a Schneider 25 mm f/0.95 lens. After the mouse was anesthetized with intraperitoneal injection of 100 uL anesthetic, the mouse was put into a light-tight chamber of the imaging system and the collection of CLI images started.

## 3. Results and Discussion

### 3.1. Results of Numerical Simulation

**Figure 3:**
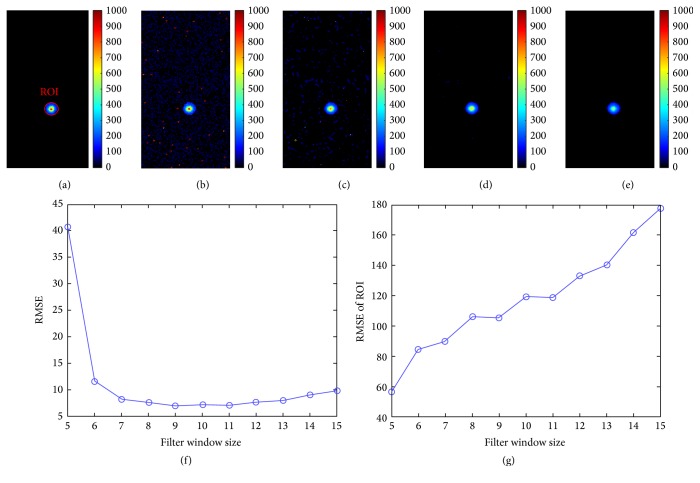
The traditional denoising method for simulated CLI. (a) Simulated CLI image. (b) Simulated CLI image with noises. (c–e) Filtered CLI images using median filter with filter window size = 5, 10, and 15. (f, g) Root mean squared error of the filtered CLI image and ROI of that.

Figure 3(a) is the simulated CL image, and Figure 3(b) is that with noises. The traditional median filter is usually used for removing these impulse noises [18, 20]. Figures 3(c)–3(e) are filtered CLI images using median filter with different filter window sizes. With the increase of filter window size, less noises remain in the filtered CLI image, but pixel values in region of interest (ROI) have more deviations. Root mean squared error (RMSE) of the filtered CLI image and ROI of that can clearly demonstrate this trend. It means that the median filter can effectively filter out impulse noises but can introduce large errors for ROI of CL signals, when large filter window size is employed. On the contrary, the median filter with small filter window can keep ROI of filtered CLI image with the same value as that of simulated CL image, but with lots of impulse noises.

**Figure 4:**
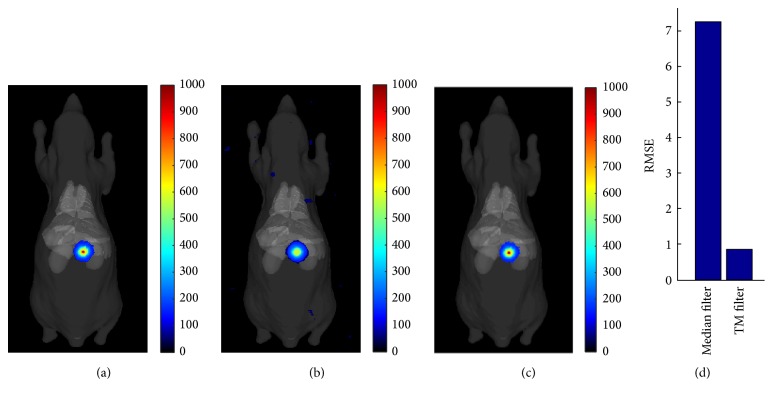
A comparison between median filter and TM filter. (a) CLI image with no noise. (b) Result of median filter. (c) Result of TM filter. (d) Comparison of RMSE for the two methods.

Figure 4 summarizes the results of median filter and TM filter for numerical simulations. Result of median filter has a few low background noises and large deviation in ROI. Quantified analysis using RMSE for the two methods has also illustrated that TM filter can acquire a high quality image for CLI.

### 3.2. Impulse Noises in CLI Image

**Figure 5:**
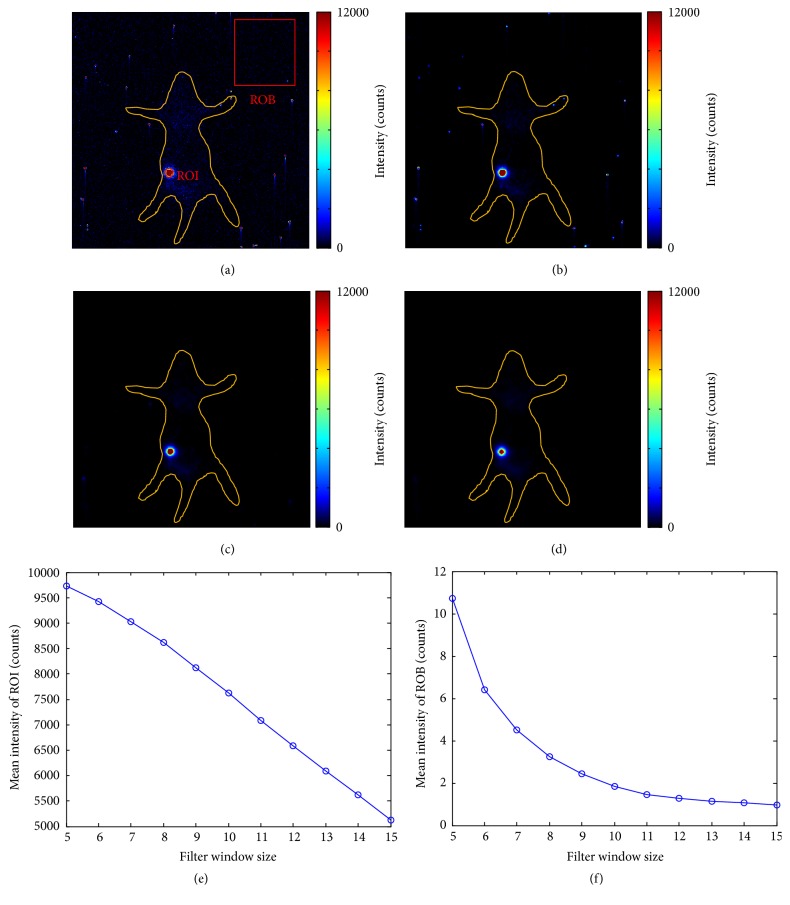
The traditional denoising method for CLI. (a) Original CLI image captured with 60 s exposure time. (b–d) Filtered CLI images using median filter with filter window size = 5, 10, and 15. A large filter window size achieves a smooth CLI image with less impulse noises. (e, f) Quantitative analysis of the relations between signal values in region of interest (ROI) marked with red circle and region of background (ROB) marked with red rectangle. The signal values in both ROI and ROB decrease with the increase of filter window size.

CL signals are so weak that a long exposure time is necessary for CLI. During the long exposure process, a great number of gamma rays may reach CCD sensor and produce heavy random impulse noises.  Figure 5(a) is an original CLI image captured with 60 s exposure time. Although ^18^F-FDG locates in region of the pseudotumor (ROI marked with red circle), lots of impulse noises intersperse among other locations in and out of the mouse without ^18^F-FDG as a result of the reach of gamma rays with arbitrary directions. Figures 5(b)–5(d) are filtered CLI images using median filter with different filter window sizes. The filtered CLI image with small filter window size contains lots of noises but the pixel values in region of signal are close to that of the original CLI image (Figure 5(b)). While a large filter window size can obtain an enough smooth filtered CLI image, the pixel values in region of signal are much smaller than that of the original CLI image (Figure 5(d)). Figures 5(e) and 5(f) are the quantified results of pixel values in ROI marked with red circle and ROB marked with red rectangle in the original CLI image.

**Figure 6:**
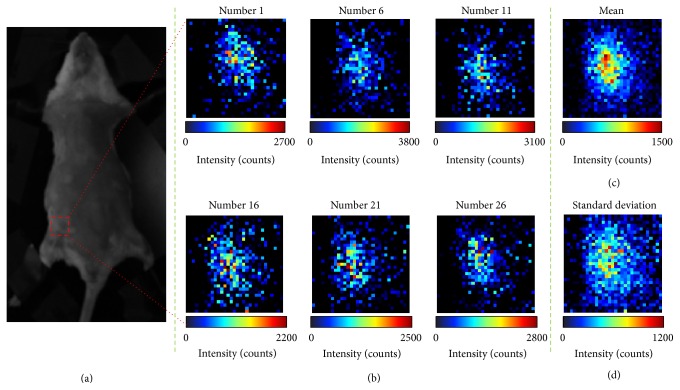
Original CLI images and mean and standard deviation of a CLI image sequence containing 30 CLI images with exposure time of 10 s. (a) is the photograph of the mouse from supine view, and the red dashed box indicates the location of pseudotumor. (b) is detailed view of red dashed box for number 1, 6, 11, 16, 21, and 26 CLI images. (c) is mean image of the CLI image sequence. (d) is the standard deviation image for the CLI image sequence.

Figure 6 shows CLI images of pseudotumor in the mouse with a short exposure time of 10 s captured at different time points. From 6 selective CLI images, we can see that the distributions of CL are generally in agreement, but differences exist in the details. The mean and standard deviation images can further illustrate these differences. The difference in capture time is not the main reason, because the interval time between each CLI image is less than 1 s. The fast gel-forming BD matrix can prevent ^18^FDG diffusing. So the high level impulse noises produce these differences. The standard deviation image also shows that the impulse noises are almost the same level as the CL signals.

### 3.3. Result of TM Filter

**Figure 7:**
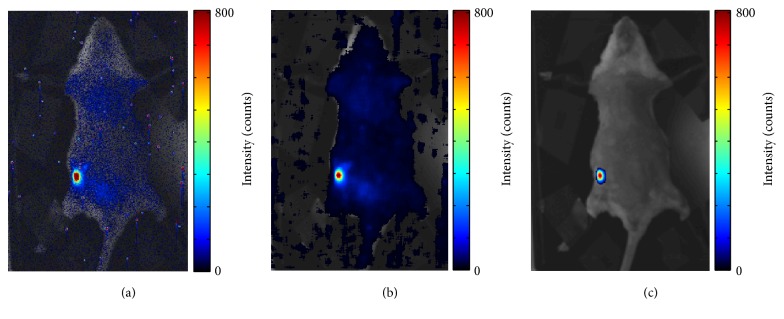
A randomly chosen original CLI image from a sequence of 30 CLI images with exposure time of 10 s for each one (a), result of median filter fused with white light image (b), and result of TM filter fused with white light image (c).

A sequence of 30 CLI images with exposure time of 10 s for each one was used to test TM filter. To compare with median filter, we randomly chose a CLI image from the sequence shown in Figure 7(a). It is clearly seen that random impulse noises induced by gamma radiation have higher intensities than that of CL signals from high ^18^FDG concentration area.  Figure 7(b) is the result of median filter, which has lots of low background noise that emerged with the smooth process of median filter. The result of TM filter almost has no noise shown in Figure 7(c).

**Figure 8:**
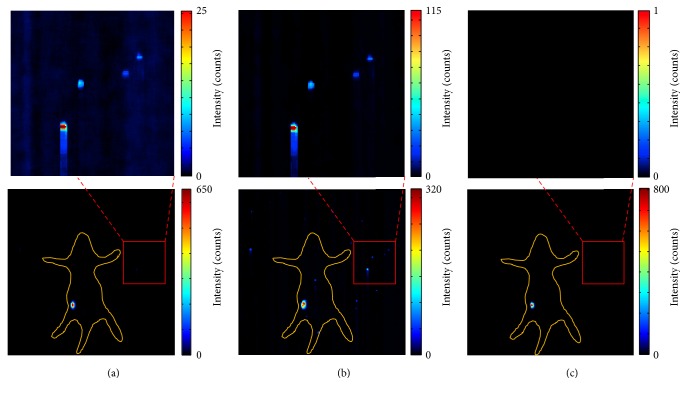
Mean image of 30 CLI images filtered using median filter (a), standard deviation image of 30 CLI images filtered using median filter (b), and filtered CLI image using TM filter (c). The top is the enlarged image corresponding to the red rectangle region.

To further illustrate noise suppression of TM filter, we investigate the background region outside of the mouse after filtering. If TM filter is effective for suppressing these impulse noises, there will be no noises in the background region of the filtered CL image. Due to median filter works on a single CLI image, we obtain mean and standard deviation images of 30 filtered CLI images using median filter as shown in Figures 8(a) and 8(b) to compare with TM filter. In Figure 8(a), there seem to be no noises in the background region after average of 30 filtered CLI images using median filter, but noises can be clearly seen in the enlarged image of the region of background marked with red rectangle. More noises emerge from the standard deviation image of 30 filtered CLI images using median filter in Figure 8(b). The filtered CLI image using TM filter in Figure 8(c) almost has no impulse noises, and particularly we find no noise in the enlarged background image.

### 3.4. Sequence Length for TM Filter

**Figure 9:**
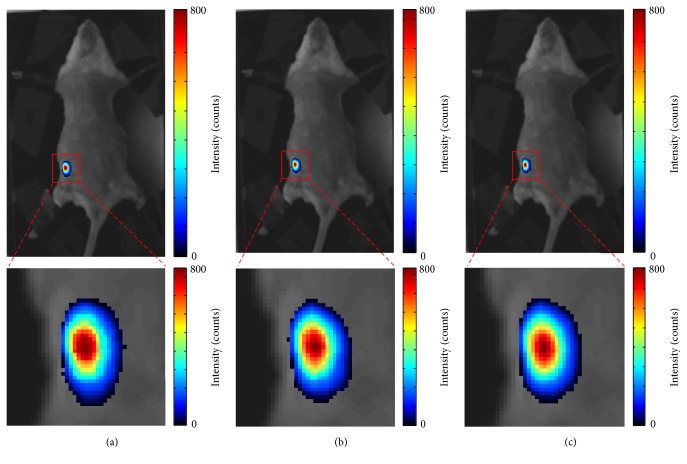
The influence of sequence length to TM filter. (a), (b), and (c) are results of TM filter for *N* = 5, *N* = 10, and *N* = 30, respectively.

Generally, a large sequence containing more CLI images has greater ability to remove random impulse noises, but that will cost more time for data acquisition.  Figure 9 shows the influence of sequence length to the TM filter. The proposed TM filter can maintain the distributions and values of CL signals even when the number of CLI images decreases to *N* = 5.

### 3.5. Exposure Time for Each CLI Image

**Figure 10:**
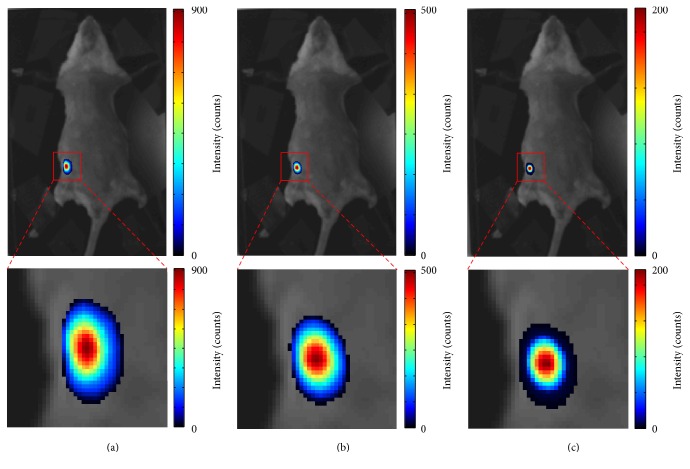
The results for TM filter with different exposure time *T*. (a), (b), and (c) are *T* = 10 s, *T* = 5 s, and *T* = 2 s, respectively.

Short exposure time is helpful in reducing the random pulse noise induced by gamma radiation, but CL signals cannot be collected using unduly short exposure time when radioactivity is rather low. In this experiment, the radioactivity of ^18^F-FDG in the pseudotumor was 100 uCi. We use different lengths of exposure time to investigate the characteristic of TM filter.  Figure 10 summarizes results of TM filter with exposure time of *T* = 10 s, *T* = 5 s, and *T* = 2 s, respectively. The intensity and area of CL signals decrease with the exposure time *T*. Even though the exposure time *T* is shortened to 2 s, TM filter can still obtain a satisfactory result.

## 4. Conclusions

In this work, a TM filter is proposed to remove the random impulse noises induced by high energy gamma rays generated from radioactive decay of medical nuclides. This method synthesizes characteristics of randomness and pulse for random pulse noises in CL images and employs a temporal median-like filter in a temporal sequence of CL images. Several simulation and in vivo experiments were presented to verify the proposed TM filter, and these results demonstrated that TM filter can effectively remove random impulse noises induced by gamma radiation and achieve a robust CL image. Several key properties of TM filter were also investigated based on in vivo experimental data to fully understand robustness and usability of this method.

In terms of extremely weak CL signal, traditional CLI collects a single CL image with minutes-long exposure time, which often contains lots of random impulse noises due to the arrival of gamma rays to CCD sensor. With the increase of the exposure time, the original CL image contains more and more impulse noises. In order to filter out these random pulse noises, traditional median filter with a large filter window size is employed. The filtered CL image of this single acquisition strategy shows instability and oversmoothness. The proposed method can overcome these drawbacks by collecting a temporal sequence of CLI images with seconds-long exposure time and a TM filter. With a multiple CLI images strategy, the proposed method is useful for suppressing these random pulse noises and getting robust CLI image. The smooth effect of TM filter is to work on pixels of CLI images collected at different time points located in the same coordinate, so the proposed method avoids smoothing CLI image on spatial domain when removing the random pulse noises. Finally, the proposed method can acquire high quality CLI images with high robustness.

To concentrate on the validity of TM for CLI, we perform an in vivo experiment based on a pseudotumor model. The nonspecific distributions of medical nuclides often make the actual CLI applications contain complicated circumstances. So the proposed method needs to be tested by different actual CLI applications.
